# Unveiling the unseen toll: exploring the impact of the Lebanese economic crisis on the health-seeking behaviors in a sample of patients with diabetes and hypertension

**DOI:** 10.1186/s12889-024-18116-6

**Published:** 2024-02-27

**Authors:** Michelle Cherfane, Myriam Boueri, Elio Issa, Racha Abdallah, Ali Hamam, Kassem Sbeity, Anthony Saad, Aniella Abi-Gerges

**Affiliations:** 1 Gilbert and Rose-Marie Chagoury School of Medicine, Lebanese American University, Byblos, P.O. Box 36, Lebanon; 2 INSPECT-LB (Institut National de Santé Publique, d’Épidémiologie Clinique et de Toxicologie- Liban), Beirut, Lebanon

**Keywords:** Economic crisis, Hypertension, Diabetes, Medications, Healthcare for all

## Abstract

**Background:**

Against the backdrop of Lebanon’s escalating multifaceted crisis which resulted in medication shortages for chronic diseases and unaffordable healthcare services, the current study endeavors to shed light on a critical yet overlooked facet of the Lebanese economic crisis– its profound impact on the health-seeking behaviors of patients with hypertension and diabetes mellitus.

**Methods:**

An exploratory cross-sectional study based on an online questionnaire was conducted on 156 adult Lebanese citizens diagnosed medically with either hypertension or diabetes. We gathered sociodemographic characteristics and healthcare-related challenges faced during the economic crisis. We also assessed stress levels using the Depression Anxiety Stress Scale (DASS-21). Descriptive and bivariate analyses were done using SPSS version 26.

**Results:**

The mean age of the population was 49.8 ± 17.7 years old, 51.6% were females and 48.4% were males, 29.7% had diabetes, 51.3% had hypertension and 19.0% had both diseases. Among all, 84.2% reported dissatisfaction with the current healthcare system, 31.6% reported changing their physician mainly because of unaffordable consultation fees (66%) or immigration of the physician (32%). Of those with hypertension and/or diabetes, less than 20% reported finding all their prescribed medications and 47% either modified or discontinued their treatment without seeking medical advice. In case of drug shortage, patients relied on stocked reserves (26%), alternative/generic medications (10%) and external sources for medication procurement such as relatives living abroad (41.7%), outsourcing suppliers (19.9%), dispensaries (19.6%) and NGOs (20.3%). All participants reported a high stress level (5.03/7) with a mean total DASS-21 score of 38.7 ± 35.8 that were attributed to August 4^th^ Beirut port explosion (81.0%), global pandemic (81%), unstable political conditions (90.5%), economic crisis (96.8%), medication shortage (91.8%) and inability to access healthcare (74.1%). Higher sub-scores for anxiety, depression and total stress were insignificantly noted in participants with both hypertension and diabetes (*p* > 0.05).

**Conclusion:**

Our findings explore how the economic crisis has taken its toll on almost all aspects of healthcare in a sample of patients with diabetes and hypertension in Lebanon. The drug shortage as well as disruptions in affordable healthcare access imposed several barriers to adequate adherence to treatment regimens and acted as important mental health stressors.

**Supplementary Information:**

The online version contains supplementary material available at 10.1186/s12889-024-18116-6.

## Background

Lebanon, once celebrated for its rich cultural tapestry and resilient society, has been grappling for nearly four years now with an unprecedented multifaceted crisis that has reverberated across all facets of life, including its healthcare landscape. The political instability along with economic and financial depression accompanied by the COVID-19 pandemic and Beirut Explosion (August 4, 2020) [1, 2] have been the major contributing factors to the crisis, with inflation rates exceeding 131.9% during the first 6 months of 2021 [3] and subsequent loss of over 81% of the original value of Lebanese Lira [4]. The burden of further economic instability on public healthcare translated into the Lebanese government restricting dollar exchange from the central bank [5] and the Ministry of Public Health (MOPH) issuing decrees to govern drug import and availability. The combined repercussions of these newly adopted decrees along with those of the roaring economic crisis, including rising unemployment, limitations on bank withdrawals and decreases in the purchasing power of people had serious effects on drug availability with a shortage of essential medications, including outpatient and inpatient cardiovascular drugs [3]. In addition, many healthcare services have been rendered unaffordable [5, 6]. This situation unveils patients with chronic diseases as a vulnerable group, mainly patients with hypertension (HTN) [7] and diabetes mellitus (DM) [8] since they comprise a significant proportion of the Lebanese population with a great burden of chronic medical care. Indeed, the prevalence of HTN in Lebanon increased from 23.1% as reported in 2005 to 31.2% in 2018 with 47% of hypertensive patients on two or more antihypertensive drugs [7, 9]. The overall national prevalence of DM is around 11% [10] as reported by the International Diabetes Federation (IDF) [8].

The interconnection between economic turmoil and healthcare outcomes has been extensively explored in the literature. Notably, Phuong et al., conducted a comprehensive analysis highlighting how drug shortages can affect patients purchasing capacity (economic outcome), mortality, drug errors, adverse drug reactions, and hospitalization changes, all known by clinical outcomes [11]. Additionally, the authors emphasized the far-reaching consequences on patient quality of life, stress, and frustration (humanistic outcomes) [11]. Moreover, other studies delved into the intricate dynamics between economic downturns and disruption in healthcare access, including dismissal of medical staff, closure of hospital sections [12], shortage of medical supplies [4], and the migration of healthcare workers, including physicians and nurses [13]. Taken together, this growing body of literature emphasizes the need for targeted investigations into the specific challenges faced by individuals managing chronic conditions in the wake of economic turmoil, especially that most of the data on medication shortage comes from industrialized countries [14–19], which may not be applied to non-industrialized ones.

In the context of Lebanon, where the healthcare system is intricately entwined with the prevailing economic conditions, understanding the implications for patients with DM and HTN is not only pertinent but also represents a critical step toward informed policymaking and targeted interventions. This exploratory study aims to contribute to this burgeoning discourse by examining the lifestyle, access to medications, health-seeking behaviors, stress levels and specific healthcare-related challenges faced by a sample of Lebanese individuals with DM and HTN, amid one of the worst economic crisis of the century.

## Methods

### Study design

We conducted an exploratory, cross-sectional study, using a snowball sample of 156 adults. Participants were recruited from the general Lebanese population during the period between December 2021-March 2022 and August 2022-November 2022 through an anonymous online survey that required on average 15–20 min to be completed. The online survey, which was created using Google Forms platform, was distributed through social media channels, such as Facebook, Instagram, and WhatsApp. Prior to starting the questionnaire, an informative introduction was included which clearly stated the purpose of the research study and highlighted essential criteria for the participants’ consent such as voluntary participation, confidentiality and anonymity of the information provided by them. The survey was accessible exclusively to Lebanese individuals aged above 18 years old, who consented to this study and reported a previous diagnosis of either HTN and/or DM. All data was kept anonymous.

### Questionnaire

The online self-administered questionnaire consisted of 81 questions that were either closed-ended or 5/7-point Likert scale questions, ranging from 1 (the lowest) to 5/7 (the highest). The first 8 questions targeted the respondents’ sociodemographic traits including a question about having any type of health insurance. The following questions mainly tackled DM (12 questions) and HTN (13 questions), assessing the history of the disease, the existence of any other comorbidities, the prescribed medications and how the economic crisis affected their medication acquisitions. The next 19 questions focused on the respondents’ health-seeking behaviors and lifestyle including their sugar and salt intake, drinking, smoking, and exercise habits. In addition, information regarding access to healthcare was gathered and their perceptions on their health, particularly with respect to clinical consequences in case of changing/stopping their medications without referring to their physicians and their inability to follow up with their physicians, was captured. The third part of the questionnaire assessed the respondents’ mental health, starting with an overall evaluation of stress level rated on a scale of 1 to 7 with 1 being the lowest and 7 being the highest. We asked about the factors contributing to stress and assessed stress in its different dimensions using the validated 21-item Depression, Anxiety and Stress Scale (DASS-21) questionnaire which serves as a quantitative assessment tool for measuring distress across the three dimensions of depression, anxiety, and stress [20, 21]. Respondents rated each of the 7 questions per axis on a scale from 0 to 3, with the scores interpreted as follows: 0 points for never (the statement did not apply to me at all), 1 point for sometimes (the statement applied to me to some degree, or some of the time), 2 points for often (the statement applied to me to a considerable degree, or a good part of the time) and 3 points for almost always (the statement applied to me very much, or most of the time). The total for each scale is multiplied by 2 and consequently, each scale score ranges from 0 to 42, with higher total scores reflecting greater severity in depression, anxiety, and stress levels. The severity levels of the different dimensions were categorized into normal, mild, moderate, severe and extremely severe, based on cutoff points previously used [20] as shown in Table 1. It is worth noting that the participants answered only the questions that applied to their diagnosis and situation.

**Table 1:** Severity levels of depression, anxiety, and stress

Level/Disorder	Depression	Anxiety	Stress
Normal	0–9	0–7	0–14
Mild	10–13	8–9	15–19
Moderate	14–20	10–14	19–25
Severe	21–27	15–19	26–33
Extremely severe	≥ 28	≥ 20	≥ 34

The contact information of the principal investigator/ corresponding author of this study and the Institutional Review Board (IRB) committee at the Lebanese American University (LAU) were also included should the respondent wish to ask questions or withdraw from the study.

The English questionnaire was translated to Arabic and back translated to English for consistency and validity by a certified bilingual translator, as previously reported [22]. The questionnaire was then pilot tested on a sample of 15 bilingual individuals. The resulting data were not included in the final analysis, but were rather used to assess clarity, readability, cultural relevance of both language versions, and estimate the needed time for questionnaire completion. Consequently, the questionnaire was edited before its online administration. The consenting participants were given the flexibility to choose between English and Arabic based on their comfort and preference; 48 (31%) filled the questionnaire in Arabic and 106 (69%) chose to fill it in English. The study was approved by the IRB committee at LAU under the code number *LAU.SOM.AG1.11/Jan/2022*.

### Statistical analysis

Data was analyzed using SPSS software version 26. First, descriptive analysis was conducted, using counts and percentages for categorical variables and means and standard deviations for continuous measures. The DASS-21 total score was computed, in addition to the three separate sub-scores related to the depression, anxiety and stress dimensions. The sample distribution was checked by visual inspection of the histogram and verified by the normality line of the regression plot and scatter plot of the residual. This was done on continuous variables. For bivariate analysis, the student t-test was used to compare two means of continuous variables between dichotomous groups or the Mann-Whitney test when appropriate. While the ANOVA was used to compare between 3 or more means (or Kruskal Wallis test in case of non-normality of the variable or non-homoscedasticity of the variances), the Chi-square test was used to compare percentages (or Fisher exact test if expected counts are lower than 5). A *p*-value < 0.05 was considered significant.

## Results

### Characteristics of the surveyed population

A total of 156 Lebanese respondents, previously diagnosed with HTN and DM, consented to participate in this study, with an average age of 49.82 ± 17.77. Of these, 80 were females (51.6%) and 75 were males (48.4%). The general characteristics of the study population are presented in Table 2. Most of the participants were from Mount Lebanon (46.8%), married (59%) and have reached high school (39.2%). Only around half of the participants are working (49.0%) and around two third of the participants (67.9%) are medically insured through either governmental insurance (28.2%) or private insurance companies (39.7%) while 32.1% are self-payers.

**Table 2:** Characteristics of the surveyed participants (*n* = 156)

Age (years): Mean ± Standard Deviation	49.82 ± 17.77	
Variable	Frequency (N)	Percent (%)
***Demographics***		
**Gender**		
Male	75	48.4
Female	80	51.6
**Region**		
Beirut	41	26.3
Mount Lebanon	73	46.8
Other	42	26.9
**Marital Status**		
Single	39	25.0
Married	92	59.0
Divorced/Separated/Widowed	25	16.0
**Education**		
High School or Less	60	39.2
Undergraduate	53	34.6
Post-Graduate	40	26.1
**Working Status**		
Retired	20	12.9
Student	10	6.5
Unemployed	49	31.6
Working	76	49.0
**Personal Current Monthly Income (1$=1,507 LBP)**		
Less than 750,000 LL	8	5.1
750,000–1,500,000 LL	11	7.1
1,500,000–3,000,000 LL	19	12.2
3,000,000–6,000,000 LL	27	17.3
More than 6,000,000 LL	26	16.7
Not applicable	51	32.7
Prefer not to answer	14	9.0
**Insurance**		
Government Insurance	44	28.2
No Insurance	50	32.1
Private	62	39.7
***Lifestyle and Health Seeking Behaviors***		
**Have you ever smoked?**		
No	71	44.9
Yes and stopped	20	12.7
Yes and still smoking	67	42.4
**Have you ever drunk alcohol?**		
No/stopped	81	51.3
Occasionally	71	44.9
Everyday	6	3.8
**Have you ever exercised? If yes, how regularly do you exercise?**		
No	108	68.4
Yes, less than 3 times/week	8	5.0
Yes, 3 times a week to everyday	42	26.6
**Do you have a physician that you visit on a regular basis?**		
No	41	25.9
Yes	117	74.1
**Are you still having regular follow-ups with a physician?**		
No	29	18.4
Yes, once/twice per year	106	67.1
Yes, every 1 to 3 months	23	14.6
**Do you still go to the same physicians before and after the crisis?**		
No	50	31.6
Yes	108	68.4
**Level of satisfaction with the current Lebanese health care system**		
Unsatisfied	133	84.2
Neutral	18	11.4
Satisfied	7	4.4

The lifestyle and health-seeking behaviors of the participants are shown in Table 2. Our findings indicate that 42.4% of the participants are current smokers, less than 5% of the participants consumed alcohol daily and approximately one third of the participants (31.6%) reported engaging in regular physical exercise. The average of the sugar and salt intake in the surveyed population was reported to be 2.65 ± 0.90 and 2.56 ± 0.97, respectively on a scale from 1 to 5 with 1 being the lowest and 5 the highest (data not shown).

**Fig. 1:**
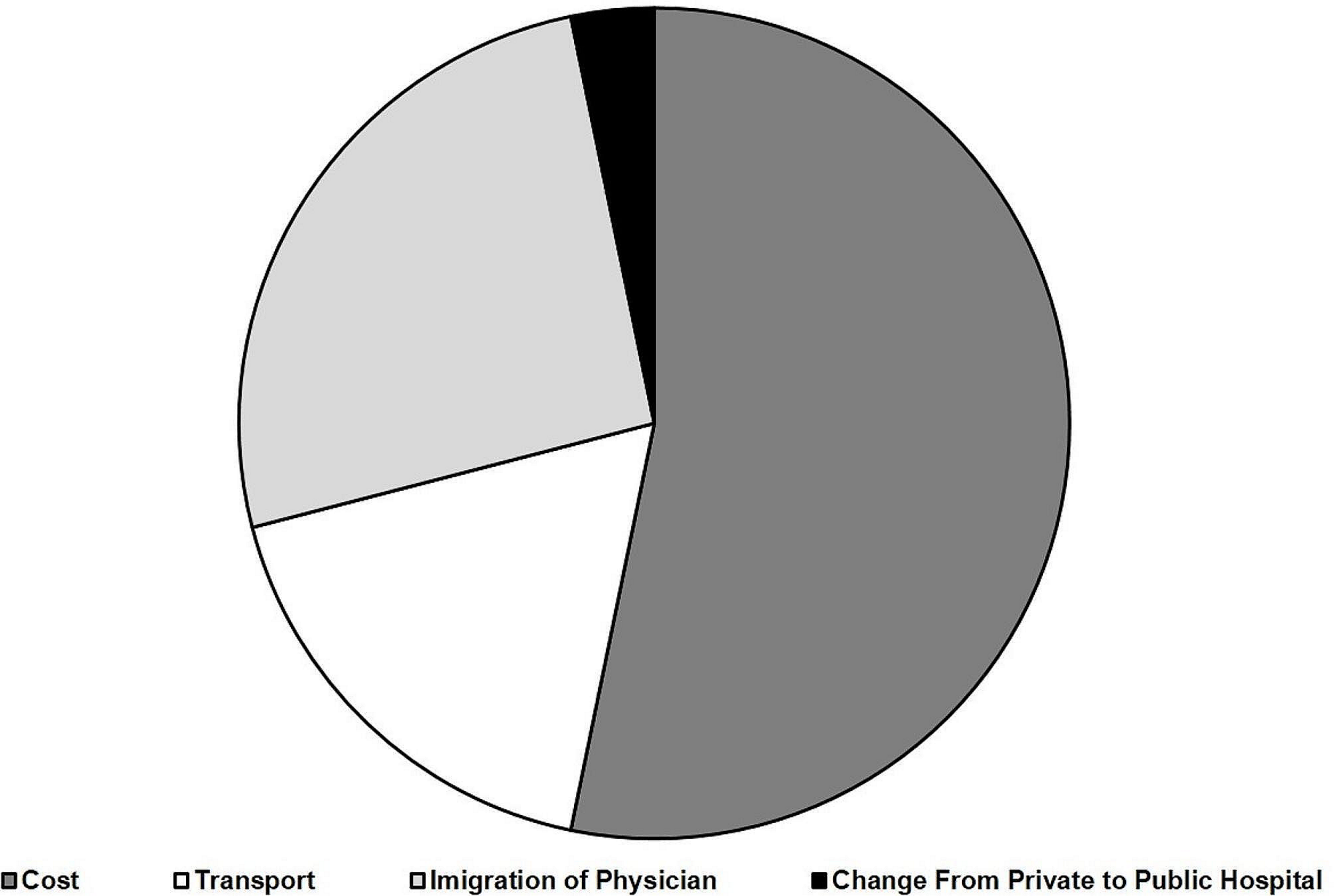
Reasons for not visiting the same treating physician

When asked about their medical follow-ups, around three quarters of the participants (74.1%) reported having a primary physician, designated as their go-to person for health services, who acted as a point of reference for their healthcare needs and monitored their health condition on a regular basis. The majority of the respondents (67.1%) scheduled follow-up visits once or twice a year while 14.6% visited their medical doctor every 1 to 3 months. Moreover, 31.6% of the participants reported not being able to visit their primary physician during the economic crisis (Table 2). The underlying reasons for this change were mainly attributed to the cost of medical consultations (66% of participants), followed by the immigration of their primary physician (32%), challenges related to transportation/access medical healthcare (22%), and transition from private to public-type healthcare providers (4.0%) (Fig. 1). Most participants (84.2%) expressed their dissatisfaction with respect to the current healthcare system in Lebanon (Table 2).

### Medication acquisition and health literacy amongst Lebanese participants with DM and HTN during the Lebanese economic crisis

Our study sample consisted of 77 participants with DM and 111 with HTN (Table 3). Moreover, 44.2% of the participants with DM and 64% with HTN presented with other co-morbidities. Some participants (19%) reported having both diseases, DM and HTN.

**Table 3:** Medication acquisition and health literacy amongst Lebanese participants with DM and HTN

	DM (*n* = 77)		HTN (*n* = 111)	
	Frequency (N)	Percent (%)	Frequency (N)	Percent (%)
**For how long have you had the disease? (years)**				
0–5	30	39.0	46	41.4
5–10	26	33.8	22	19.8
10+	21	27.2	43	38.8
**How regularly do you currently check your fasting blood glucose/blood pressure?**				
Daily-Weekly	49	63.6	62	55.9
Monthly-Never	28	36.4	49	44.1
**Do you have other comorbidities?**				
No	43	55.8	40	36.0
Yes	34	44.2	71	64.0
**How many prescribed medications are you taking currently?**				
0	2	2.6	1	0.9
1–2	36	46.8	59	53.2
3+	39	50.6	51	45.9
**Have you been able to find the medications that have been prescribed to you?**				
No, none of them	12	15.6	15	13.5
Yes, all of them	12	15.6	20	18.0
Yes, some of them	53	68.8	76	68.5
**Have you been able to afford the medications prescribed to you?**				
No	39	50.6	46	41.4
Yes	38	49.4	65	58.6
**Have you changed the medication, reduced the dosage, or stopped taking the drug without medical supervision/advice?**				
No	36	46.8	65	58.6
Yes	41	53.2	46	41.4
**Do you think that there are any side effects of changing the dosage without referring to the physician?**				
I do not know	14	18.2	20	18.0
No	7	9.1	6	5.4
Yes	56	72.7	85	76.6
**Perceived stress score (0–7)**	5.14 +/- 1.4 [1–7]		4.92 +/- 1.6 [1–7]	

N.B. Some participants in each of these categories are with both diabetes and hypertension simultaneously

The onset of DM among participants was reported to have occurred mostly during the last five years (39% of the participants), followed by the 5 to 10 years interval (33.8%), while 27.2% were diagnosed since more than 10 years. Most participants with DM (63.6%) reported monitoring their blood glucose levels either daily or weekly during the economic crisis, while 36.4% either checked their glycemia monthly or have never measured it. Additionally, most of the respondents (97.4%) were taking one or more prescribed antihyperglycemic medications. Limited availability of antihyperglycemic medications during the economic crisis was reported by 15.6% of the participants with DM, while the remaining participants had access to at least one of their medications. Nearly half of the participants with DM (49.4%) had trouble affording the cost of their medications. Amidst this situation, more than half (53.2%) of the participants had to make unsupervised medical decisions, including changes in medications, dosages, or even discontinuation of medications. When they were not able to find their prescribed drugs, the majority took a generic. Approximately three-quarters of the participants with DM believed these unsupervised medical decisions carry side effects on health (72.7%) (Table 3).

Among the participants diagnosed with HTN, a slightly bimodal distribution of the disease duration since diagnosis was observed. More than 50% of participants monitored their blood pressure on a daily or weekly basis. Notably, only 15.9% of participants exhibited uncontrolled readings (> 140/90 mmHg) at the time of data collection. Most of the participants (99.1%) were on one or more prescribed antihypertensive medications. Access to most of the prescribed medications was generally available to participants with HTN, except for 13.5% who faced challenges in procuring antihypertensive medications. The cost of the prescribed medications was reported to be a limiting factor by 58.6% of the respondents diagnosed with HTN. Moreover, 41.4% of participants either modified, reduced the dosage, or discontinued their prescribed medications without seeking medical advice. When they were not able to find their prescribed drugs, the majority took a generic. Additionally, a considerable proportion (76.6%) believed that their decisions impose potential side effects as a result (Table 3).

**Fig. 2:**
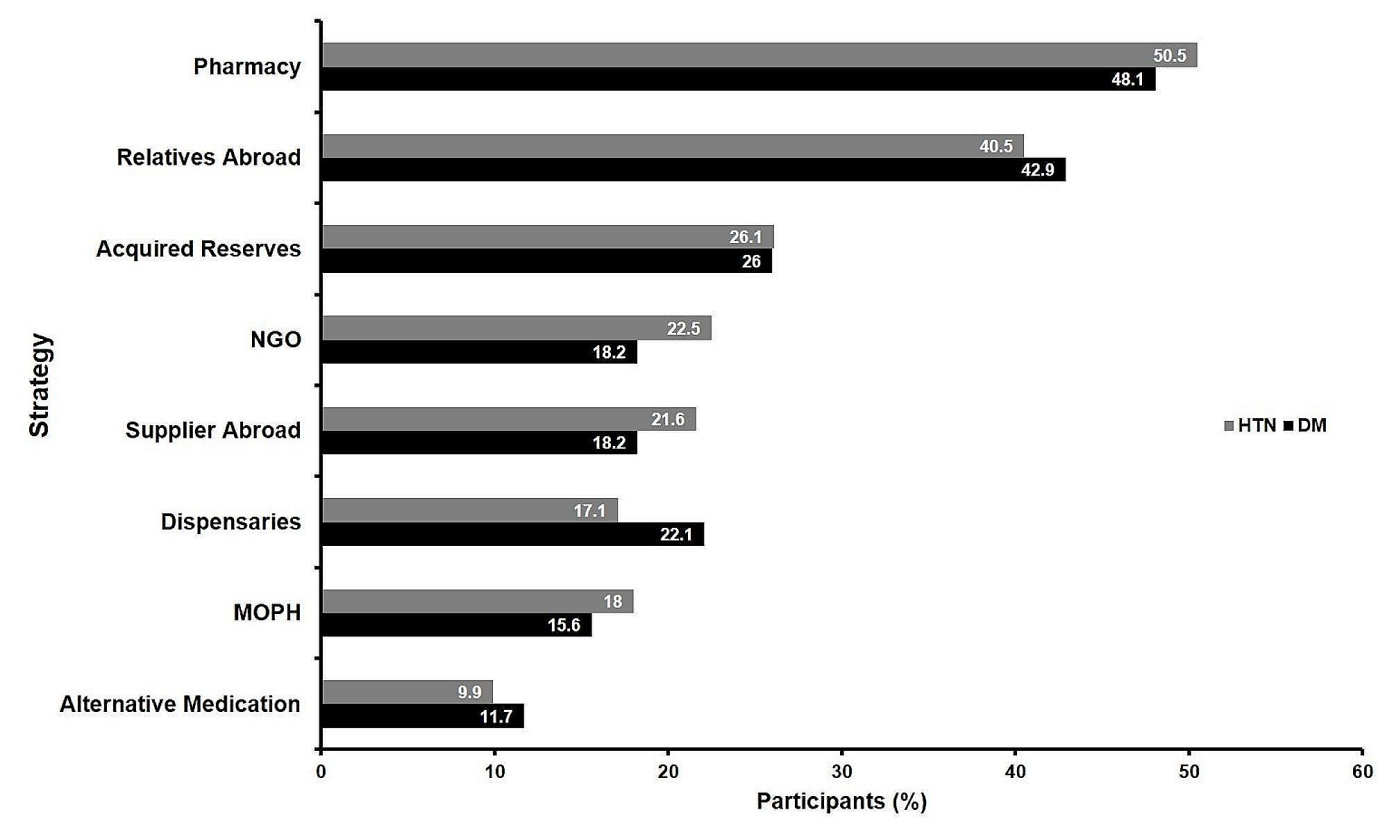
Strategies employed by participants to acquire their medications amidst the current Lebanese economic crisis. HTN: hypertension; DM: diabetes mellitus; NGO: non-governmental organization; MOPH: ministry of public health

Faced with the economic crisis, pharmacies remained a primary provider of medications for participants in our study sample (DM: 48.1%; HTN: 50.5%). Nonetheless, procurement of medications from external sources outside Lebanon, either relatives (DM: 42.9%; HTN: 40.5%) or suppliers abroad (DM: 18.2%; HTN: 21.6%), was a notable method used by the participants. Other strategies for drug acquisition included nongovernmental organizations (DM: 18.2%; HTN: 22.5%), dispensaries (DM: 22.1%; HTN: 17.1%), and the MOPH (DM: 15.6%; HTN: 18.0%). Furthermore, some participants relied on stored reserves of their medications (DM: 26.0%; HTN: 26.1%), while others switched to an alternative medication (DM: 11.7%; HTN: 9.9%) (Fig. 2).

**Fig. 3:**
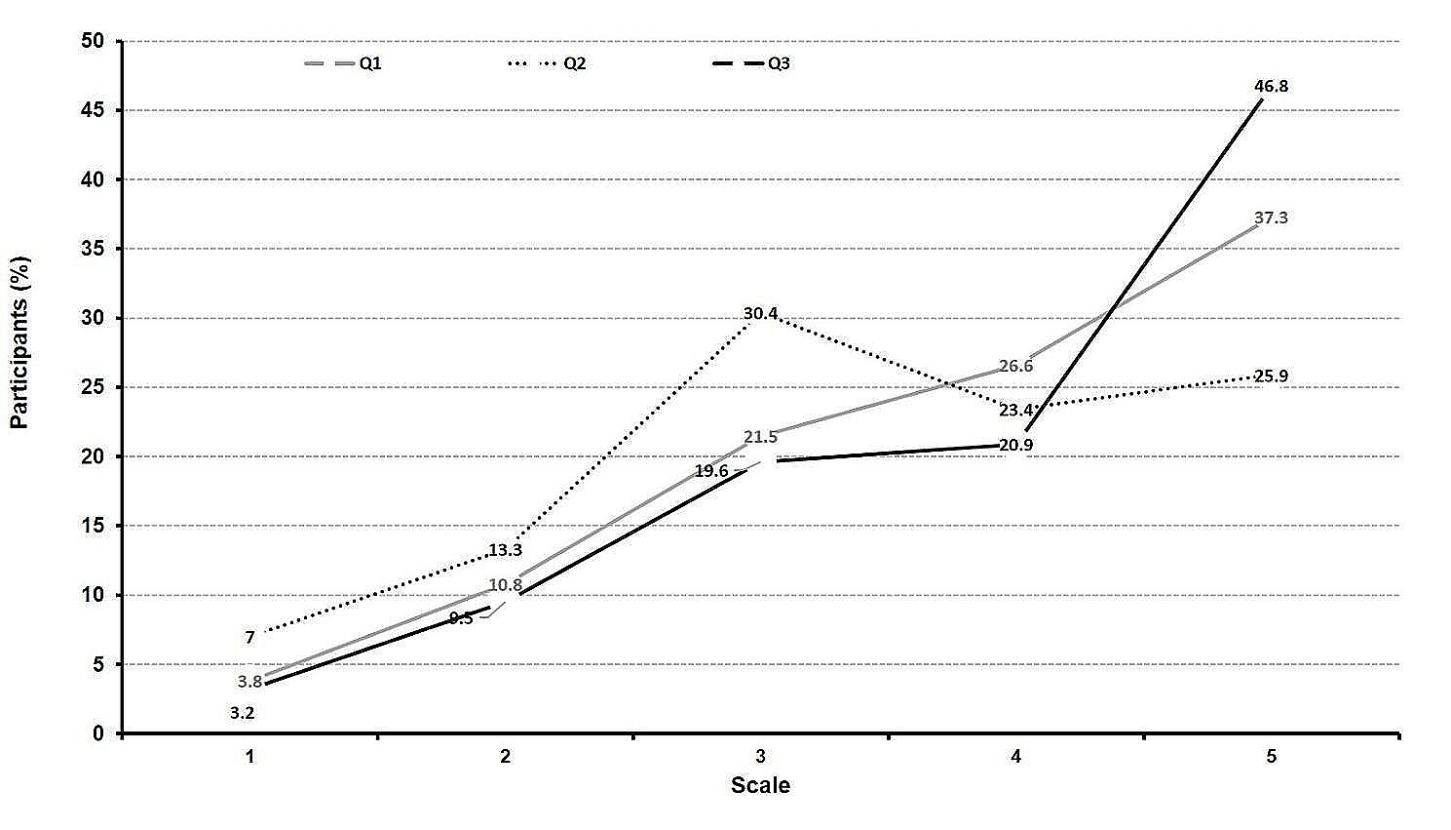
Participants’ reported perception toward their health, measured on a scale from 0 (not much) to 5 (a lot). Q1: how seriously will your health be affected if you stop your medication without referring to the physician. Q2: how seriously will your health be affected if you cannot have regular health check-ups. Q3: how seriously will your health be affected if you cannot be hospitalized when the need arises

The percentage of patients evaluating their perception toward their health, on a scale of 0 to 5, specifically with the context of medication shortage and a lack of access to healthcare is illustrated in Fig. 3. Subsequently, the mean score (SD) reported by all participants when asked if their health will be affected if they stop taking their medications without medical referrals, stop having regular health checkups and if they cannot be hospitalized when needed are 3.83 (1.15), 3.48 (1.2) and 3.99 (1.15), respectively. In addition, 52% of the participants thought that drug shortage could lead to hospitalization, while 22% thought that it was unlikely and 26% were neutral to that matter. Moreover, 30.4% of the participants believed that drug shortage can lead to death, *vs*. 25.9% who disagreed with this statement and 36.1% who did not know. Furthermore, 74.1% of the participants agreed that changing medication dosages without referring to their physician could seriously affect their health, compared with 12% who disagreed with this statement and 29% who held a neutral opinion on this issue.

### Assessment of mental health in the participants with DM and HTN during the Lebanese economic crisis

The mean score (SD) on stress level reported by participants was 5.03 (1.45) with DM and HTN individuals exhibiting comparable stress scores (SD) of 5.14 (1.4) and 4.92 (1.6), respectively (Table 3). Participants attributed their stress to the economic crisis (96.8%), medication shortage (91.8%), political instability in the country (90.5%), the august 4^th^ Beirut port explosion (81.0% of participants), global pandemic (81%) and inability to access healthcare (74.1%).

In addition, the mental health of respondents at this point in time was assessed using the DASS-21 questionnaire (Table S1, Supplementary materials). DASS-21 score (SD) in all participants was 38.7 (35.8). Table 4 shows the mean score (SD) on each of the depression, anxiety, and stress dimensions as well as the total DASS-21 score.

**Table 4:** Difference in the mean depression, anxiety, stress, and overall DASS scores among participants

Group	DASS Anxiety		DASS Depression		DASS Stress		DASS Total	
	Mean ± SD	*P*-value	Mean ± SD	*P*-value	Mean ± SD	*P*-value	Mean ± SD	*P*-value
DM only	9.8 ± 10.1	0.056	11.4 ± 11.2	0.077	13.5 ± 10.7	0.089	34.7 ± 30.6	0.065
HTN only	11.0 ± 11.7		12.2 ± 12.1		12.8 ± 11.8		36.0 ± 34.5	
DM + HTN	16.2 ± 14.1		17.7 ± 15.5		18.5 ± 15.4		52.4 ± 43.7	

*P-value < 0.05

Overall, there was a gradual subsequent increase in the anxiety, depression, and stress levels, in patients with either DM, HTN or both diseases. In other words, among the three dimensions, stress had the highest score in all groups and in all the dimensions. Participants with both DM and HTN exhibited higher mean scores of anxiety, depression and stress compared to having either of the diseases, but the difference was non-significant. Moreover, this was seen in the overall score where patients with both diseases had a mean (SD) score of 52.4 (43.7) compared to 34.7 (30.6) and 36.0 (34.5) for those with DM or HTN, respectively (*p* > 0.05 for all). However, when comparing mean (SD) scores of individuals with both diseases to either having DM or HTN, the difference reached statistical significance in the total and all sub-sets of the DASS-21 score, except for the stress level and in patients with both diseases compared to having DM only (9.27 (7.7) *vs*. 6.74 (5.36); *p* = 0.081).

**Fig. 4:**
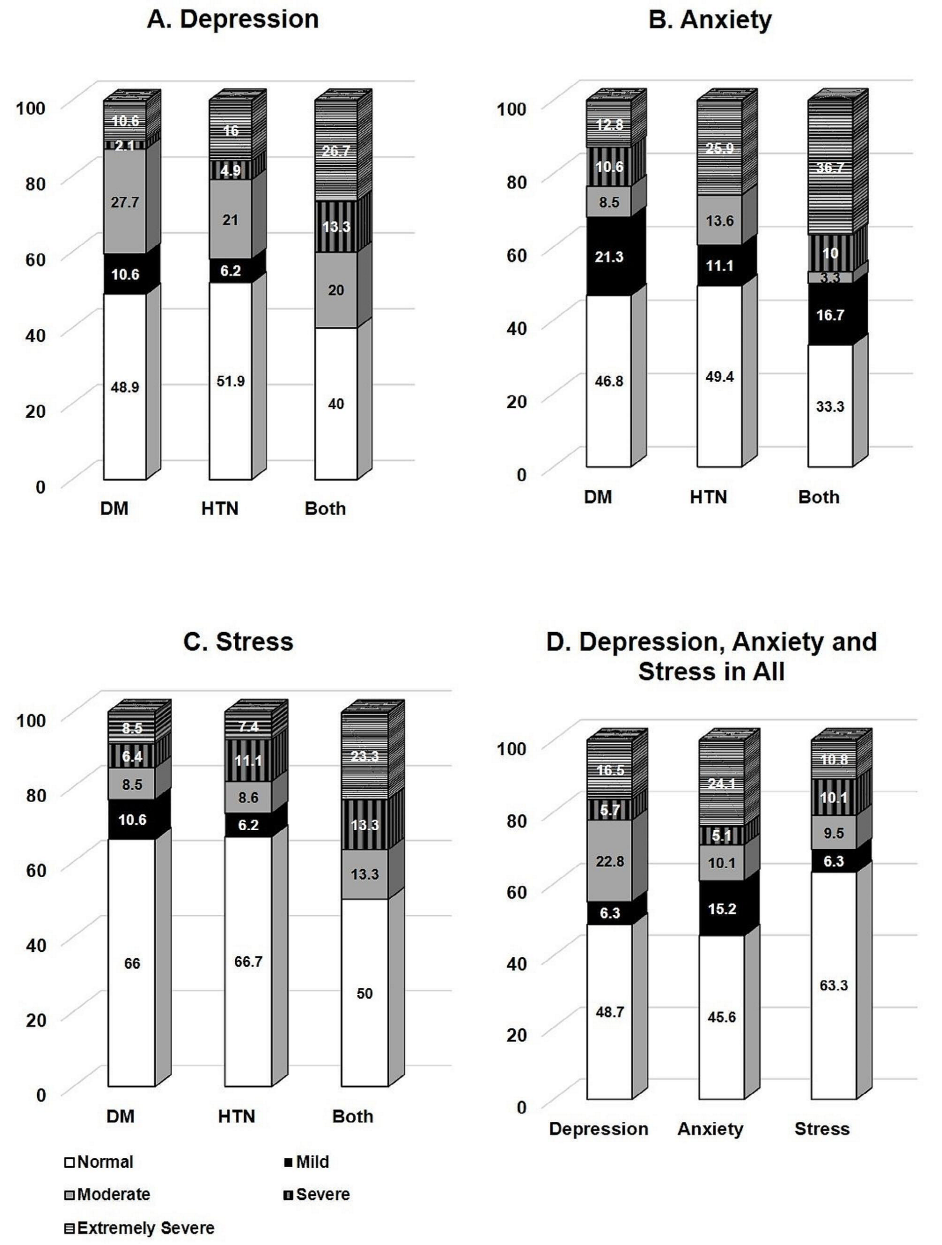
Severity of Depression, Anxiety and Stress in all participants and stratified by disease. *p* > 0.05 for depression and stress using Fisher exact test; *p* = 0.007 for anxiety using Fisher exact test; *p* < 0.01 for all using McNemar test

The difference in the severity of depression, anxiety, and stress in all participants, and comparatively in patients with either DM, HTN or both is further illustrated in Fig. 4, which shows that a higher proportion of participants with both diseases had extremely severe depression (Fig. 4A), anxiety (Fig. 4B) and stress levels (Fig. 4C). For example, 36.7% of those with both diseases had extremely severe anxiety compared to 25.9% of those with HTN and 12.8% of those with DM (*p* = 0.007). In addition, 24.1% of all participants had extremely severe anxiety compared to 16.5% had extreme depression and 10.8% had extreme stress (*p* < 0.01).

## Discussion

The study results explored how the economic crisis has taken its toll on the healthcare using a sample of Lebanese respondents who reported being previously diagnosed with DM, HTN or both. The devaluation of the national currency has led to a surge in medication prices, drug shortage and disruptions in affordable healthcare access. Amidst these challenges, barriers to adequate adherence to treatment regimens, high reported stress levels and unsatisfaction with the current Lebanese healthcare system were reported in participants with DM and HTN.

In our study sample, there is a greater representation of Mount Lebanon and the capital, Beirut, compared to other remote regions, which may point to regional differences in socioeconomic conditions, infrastructure, and access to healthcare that may have an impact on health outcomes [23]. Indeed, the impacts of the economic crisis were shown to be more pronounced in rural areas where people have lower incomes and decreased access to healthcare services [24, 25]. A study conducted in rural Crete showed that most patients, without referring to their physicians and to face the high costs, lowered their medication doses [26]. This was specifically prominent in chronic disease patients where insulin users reduced the quantity used. Moreover, the study sample presents a high prevalence of both married participants and respondents with undergraduate/postgraduate degrees. Marital status and education have been shown to correlate with improved clinical outcomes. Married people were reported to have access to resources and social support that have a positive impact on their health [27]. Education level may have an impact on health literacy, health-seeking behavior, and knowledge of health [28].

The wide range of job situations and income levels reported by the participants reflect the country’s economic inequalities. It is noteworthy that only 34% of the participants reported an income higher than 3, 000, 000 LBP and 32.1% had no medical insurance. Economic hardship makes it difficult for patients to pay for medical treatment and prescription drugs, thus worsening clinical outcomes and leading to health disparities. This association has been cited by different studies [29, 30] where lower income levels and unemployment were associated with poorer health status and less access to healthcare services. The Lebanese economic situation not only affected drug costs and availability but also led to non-adherence to treatment and medical follow-ups, increases in consultation fees and a significant emigration of many physicians. These challenges led a high proportion of the Lebanese respondents suffering from DM and HTN to change their primary care physician or to switch from private to public institutions, which may lead to fragmented care and jeopardize treatment continuity [31]. Overall, most of the participants were unsatisfied with the current Lebanese Healthcare system. Our data are in agreement with results from Greece where the concomitant rise in unemployment and decreases in the purchasing power of the public led to a decrease in utilization of private care as opposed to public care, thus increasing the burden on an already strained public sector [32].

More than 85% of the medications in Lebanon are imported [33]. Disruptions within the pharmaceutical supply chain due to the economic crisis [34] left patients with DM and HTN with a few drugs locally produced. This has contributed to the scarcity of DM and HTN medications, thereby affecting patient’s treatment continuity [34]. To alleviate the burdens caused by drug shortages, the Lebanese MOPH issued several decrees regarding the import and pricing of drugs, the most important of which was decree 892–2021 issued on July 16, 2021, which stated that some drugs are no longer supported by the central bank. As a result, the prices of drugs increased at least four to six times. The effect of these decrees remains questionable as the efforts taken were not to the magnitude of the pre-crisis period and drug shortages, especially of antidiabetics and antihypertensives, continued. Consequently, Lebanese patients adopted several coping mechanisms to navigate the challenges of obtaining medications during economic hardships depending on their different rational. A large proportion of participants with DM and HTN (around 50%) reported challenges to access at least one of their medications with most patients reporting a change in their medication or regimen as an alternative, without referral to medical advice. In turn, this pushed some of the patients to discontinue their medication because of their inability to afford either their cost or the medical consultation fee to address the issue or even because they do not perceive a detrimental effect on health if they stop. Other reduced the dosage, or altered the regimen so that a box of medication can be used for a longer period, hence lengthening the time to buy another box. A proportion of patients sought alternative or generic, usually the available and the cheaper. Conversely, most of the patients (around 70%) employed these adaptive strategies although they were aware of the side effects of such practice on their health outcomes. This scenario is reminiscent of experiences in other nations, where decreases in the use of medications due to economic hardships were also observed with subtle differences between countries. In Greece, pharmaceutical expenditure has long been high mainly due to low use of generics and usage of new expensive medications [17]. Following the Greek economic instability that strained healthcare access, a reduction in pharmaceutical prices was imposed by the government as a corrective measure; however, this created a huge shortage in drugs in the country [35] which led several diabetic patients to refuse the usage of expensive medications and to decrease the frequency of taking their drugs [26, 36]. Needles, even though provided freely, were not of sufficient quantities for multi-injection patients further leading to a decrease in adherence [36]. The same applies to test strips used in self-monitoring of blood sugar [36]. Similarly, in Venezuela, shortage of medications have reduced access to treatment, thus leading to selling antimalarial drugs in black markets for patients at unaffordable costs for many people [19]. Moreover, in Portugal, some patients requested cheaper medications from their physicians as they could no longer afford them, whereas others cut medicine doses on their own [37] or even discontinued their treatment [38]. This was accompanied by financial pressure on pharmacies that have struggled in supplying some medications which led to shortages of medicines [37]. In Italy, patients reported that they choose pharmaceutical items based on the recommendation of a family member or friend, rather than a medical expert [39]. This could be attributed to a lack of trust in health professionals and a desire to find a simpler and less expensive option than what doctors recommend [39]. Also, the decrease in the usage of all types of drugs was prominent in low socioeconomic areas where the unemployment rate was high [38]. Continuing along with this trend, in the United States, chronically ill patients felt the financial upheaval as they were unable to purchase their prescription medications [18]. Taken together, this growing body of literature emphasizes the vulnerability of healthcare systems during economic downturns and sheds lights on the implications for patients managing chronic diseases.

The association between adherence to medications and a decrease in complications and mortality [40] in both DM and HTN [41] is well established. In Lebanon, after the shortage of outpatient as well as inpatient cardiovascular medications, increases in the incidence of decompensated heart failure, myocardial infarction, and unstable arrhythmias requiring emergency intervention were reported [3].

Although Lebanese pharmacies remained the main source of medication, a high proportion of participants straining under the weight of the economic crisis, employed adaptive strategies to ensure the prescribed medications, such as resorting to dispensaries, NGOs, online purchasing from abroad, personal import of medications, seeking alternative treatments and relying on support networks and relatives living abroad. A good proportion of individuals have stocked medications. This practice has further worsened medication shortage and unavailability in pharmacies, because there was a trend that patients with chronic diseases have stocked medications for months, especially with the projections in increased lira inflation and skyrocket increase in medication prices. In other words, people bought excess amount of medication at lower prices and stocked them in anticipation of increased prices and medication shortage. This acted as a factor in furthering the lack of medication availability in pharmacies. Taken together the abovementioned results are in accordance with earlier research done in settings with limited resources, emphasizing the necessity for a variety of strategies to guarantee drug accessibility and affordability under trying conditions [42]. However, many concerns have yet to be addressed. The major question is whether these alternative sources helped maintain an adequate level of adherence to treatment. Another concern is the long-term sustainability of such an alternative method, especially if the economic situation does not stabilize soon. Hence, further studies are needed in this regard.

Despite the Lebanese economic challenges, more than half of the participants were still able to monitor their blood sugar and blood pressure. This provides a sigh of relief, as disease monitoring is a cornerstone of management. Home glucose monitoring has been associated with improved glycemic control and reduced long-term complications from DM. Similarly, home blood pressure levels can predict target organ damage and cardiovascular outcomes better than office values [43]. In essence, discussing these specific aspects within the Lebanese context provides a nuanced understanding of how the economic crisis uniquely affects health-seeking behavior of- and medication availability for DM and HTN patients. Economic recessions were reported to disproportionately affect vulnerable populations, exacerbating health inequalities [44]. In this intricate landscape, patient coping strategies and the support of Lebanese diaspora, discussed above, emerge as a critical area in our study.

Poor lifestyle factors have been discussed in the literature particularly during the pandemic [45]. Consistently with this data, our findings show high prevalence of smoking and low prevalence of exercise.

Our study presents a humanistic dimension by shedding the lights on the mental health of patients with DM and HTN. The stress level reported by the respondents was relatively high and was attributed mainly to the Lebanese economic crisis, medication shortage, the political instability in the country, August 4^th^ Beirut port explosion, COVID-19 pandemic, and the inability to access healthcare. While several countries suffered economic turmoil, the situation in Lebanon is peculiar since its population has witnessed a combination of hardships at the same time. The reported level of stress was further explored by the DASS-21 questionnaire. The challenging times have led to extreme severe levels of anxiety, depression, and stress in all participants. Interestingly, patients with DM and HTN had higher levels of depression, anxiety, and stress, evident by higher means (SD) scores, than individuals having either of the diseases. Moreover, a higher proportion of participants with severe or extremely severe depression, anxiety and stress was noted in patients with DM and HTN compared to individuals having either of the diseases. Additionally, although a higher score was obtained for stress, a higher proportion of patients exhibited anxiety as compared to depression or stress. This can be explained by the many factors experienced by Lebanese people raising anxiety, such as instability, fear of the unknown in a chaotic environment, unclear timeline for improvement of the situation, inability to forecast the future and the probability of sudden disastrous events to be repeated (such as Beirut explosion), lack of access to hospitals (because of their destruction with the explosion), unavailability of physicians and medication shortage, etc. Importantly, a high proportion of individuals agreed that their health can be affected if they stop their medication or cannot have regular checkups or cannot be hospitalized. This could be also aligned with results of severe and extremely severe anxiety, which highlight the participants’ perception to the impact on their health in the context of lack of access to care and medication shortage. It is worth noting that the large SD that we recorded from the DASS-21 results can be attributed to the small sample size. This also indicates that the data points in our dataset are spread out over a wider range from the mean. In other words, this is suggestive of a significant amount of variability or dispersion in the data, which has led to a less consistent or more variable level of depression, anxiety, and stress with lower values than what the true mean is. Overall, our findings are in accordance with other studies reporting that shortage of medication has negative consequences on the mental and psychological aspects of the patient, including frustrations, anger, feeling like a burden to themselves and caregivers, panic, overthinking, suicide thoughts, and anxiety status.

This research study presents several limitations and strengths. We first acknowledge the small study sample size which is not representative of the entire Lebanese population. However, our exploratory findings about a small group of Lebanese patients with DM and HTN still provide a comprehensive, relevant and insightful report which captured the challenges faced by a vulnerable population in a critical point in time during the economic turmoil in Lebanon. Additionally, this is the first study that delves into the understudied domain of healthcare, specifically examining how the economic downturn in Lebanon has critically influenced the availability and accessibility of medications vital for patients diagnosed with DM and HTN and who are also strained by several other stressors in a critical point in time. As the nation faces unprecedented challenges, the ramifications on the health and well-being of individuals managing chronic conditions underscore the urgent need to comprehend and address the intricate interplay between economic hardships and healthcare outcomes. As an inherent limitation, the surveyed population was recruited through a convenient sample using a snowball method [22], which again may not reflect the general Lebanese population with DM and HTN. Besides, the distribution of the online questionnaire through social media may be limited to those with internet access, which could explain the poor response rate. However, using an online survey and a snowball method made it possible to collect data during hardships in a short time, with limited resources, and to attain a vulnerable population that is somehow difficult to reach in-person during unprecedented times of multiple crises. Our focus on understanding the health-seeking behaviors of patients with DM and HTN and the use of a descriptive methodology provided a comprehensive overview of the healthcare situation and drug availability in Lebanon. Hence, our findings should be considered as exploratory since they did not aim to identify or verify causal statistical relationships. Moreover, this exploratory/descriptive approach highlighted the impact of economic crises on healthcare outcomes, helped identify relevant topics to be explored in future research, and served as a clarion call for evidence-based policies and interventions tailored to the unique challenges faced by Lebanon. Furthermore, we acknowledge the simplicity of the statistical analysis that did not allow us to explore interactions that are more specific; the sample size further reduced the opportunities for subgroup analyses. The emphasis though is on generating insights and understanding patterns rather than on statistical precision. Additionally, the adopted questionnaire might not have assessed all aspects of economic, clinical, and humanistic outcomes of Lebanese patients with DM and HTN. It is also noteworthy that the time interval during which the data was collected plays a crucial role in the interpretation of our findings. In other words, our study describes the situation in Lebanon at a particular point in time where the population was strained by several stressors and hardships. Other factors and different events could have influenced at other times. Finally, this study is based on a survey where the participants are self-reporting data that relies on the memory and recall of the respondents. The latter can be influenced by various factors, such as time, context, emotions, motivation, and social norms.

## Conclusion

This exploratory research not only contributes to the broader discourse on the impact of economic crises on healthcare outcomes but also serves as a clarion call for evidence-based policies and interventions tailored to the unique challenges faced by Lebanon. By amalgamating insights from the existing literature with the specific nuances of the Lebanese context, this study aimed to unravel the complexities surrounding medication availability for patients managing DM and HTN in a nation at the intersection of economic turmoil and healthcare vulnerability. The chaotic environment, unstable political situation and devaluation of the national currency are still not resolved and can have further detrimental effect on the health and medical outcomes of patients with chronic diseases. It is well established that medication non-compliance can lead to uncontrolled HTN that in turn can lead to complications such as myocardial infarction, stroke, and heart failure. Similarly, uncontrolled DM can increase the risk of diabetes related complications. Patients with both diseases are at the highest risk of these complications. Future studies should consider the influence of medication shortages on the clinical outcomes of patients with DM and HTN.

### Electronic supplementary material

Below is the link to the electronic supplementary material.

Supplementary Material 1

## Data Availability

The datasets used and/or analyzed for this study are available from the corresponding author upon reasonable request.

## Abbreviations

DASS-21: Depression Anxiety Stress Scale
HTN: hypertension
DM: diabetes mellitus
LAU: Lebanese American University
SOM: School of Medicine
IRB: Institutional Review Board
NGO: Nongovernmental Organization
MoH: Ministry of Health
*vs*: versus
