# Supplementary material for: Unveiling the unseen toll: exploring the impact of the Lebanese economic crisis on the health-seeking behaviors in a sample of patients with diabetes and hypertension
Source: BMC Public Health. 2024 Feb 27;24:628. doi: 10.1186/s12889-024-18116-6 (PMC10900622; doi:10.1186/s12889-024-18116-6)
Supplement: Supplementary file 1 — Supplementary Material 1 [file 12889_2024_18116_MOESM1_ESM.pdf]

Table S1. Effect of the Lebanese economic crisis on the mental health based on the DASS-21 scale. N: frequency; %: percentage.

	**How Much the Statement Applied to You over the Past Week**							
	**Did not apply to me at all**		**Applied to me to some degree, or some of the time**		**Applied to me to a considerable degree, or a good part of time**		**Applied to me very much, or most of the time**	
	N	%	N	%	N	%	N	%
1. I found it hard to wind down	62	39.2	52	32.9	22	13.9	22	13.9
2. I was aware of dryness of my mouth	75	47.5	46	29.1	23	14.6	14	8.9
3. I couldn't seem to experience any positive feeling at all	71	44.9	48	30.4	14	8.9	25	15.8
4. I experienced breathing difficulty (eg, excessively rapid breathing, breathlessness in the absence of physical exertion)	79	50.0	40	25.4	24	15.2	15	9.5
5. I found it difficult to work up the initiative to do things	66	41.8	51	32.3	21	13.3	20	12.7
6. I tended to over-react to situations	63	39.9	53	33.5	19	12.0	23	14.6
7. I experienced trembling (eg, in the hands)	92	58.2	36	22.8	14	8.9	16	10.1
8. I felt that I was using a lot of nervous energy	57	36.1	47	29.7	34	21.5	20	12.7
9. I was worried about situations in which I might panic and make a fool of myself	85	53.8	41	25.9	16	10.1	16	10.1
10. I felt that I had nothing to look forward to	79	50.0	40	25.3	18	11.4	21	13.3
11. I found myself getting agitated	68	43.0	54	34.2	21	13.3	15	9.5

12. I found it difficult to relax	61	38.6	57	36.1	20	12.7	20	12.7
13. I felt down-hearted and blue	57	36.1	54	34.2	20	12.7	27	17.1
14. I was intolerant of anything that kept me from getting on with what I was doing	73	46.2	44	27.8	22	13.9	19	12.0
15. I felt I was close to panic	83	52.5	38	24.1	22	13.9	15	9.5
16. I was unable to become enthusiastic about anything	67	42.4	46	29.1	25	15.8	20	12.7
17. I felt I wasn't worth much as a person	84	53.2	34	21.5	19	12.0	21	13.3
18. I felt that I was rather touchy/emotional	55	34.8	54	34.2	27	17.1	22	13.9
19. I was aware of the action of my heart in the absence of physical exertion (eg, sense of heart rate increase, heart missing a beat)	64	40.5	49	31.0	22	13.9	23	14.6
20. I felt scared without any good reason	79	50.0	45	28.5	16	10.1	18	11.4
21. I felt that life was meaningless	88	55.7	40	25.3	12	7.6	18	11.4
